# Supplementary figures and images for: Celecoxib treatment alleviates cerebral injury in a rat model of post-traumatic epilepsy
Source: PeerJ. 2023 Dec 6;11:e16555. doi: 10.7717/peerj.16555 (PMC10710164; doi:10.7717/peerj.16555)

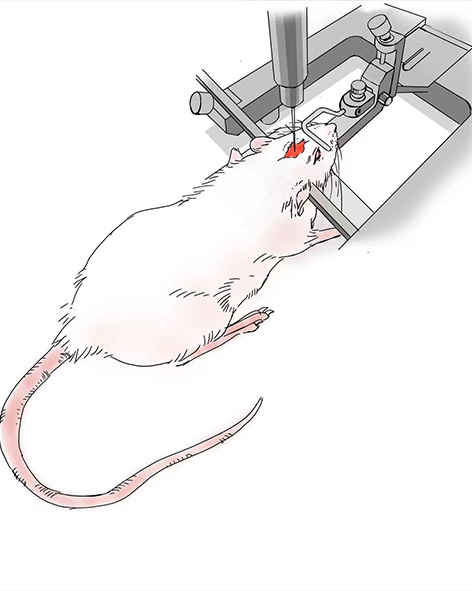

Supplement: Supplemental Information 2 [file peerj-11-16555-s002.zip › figure 1/A.jpg]

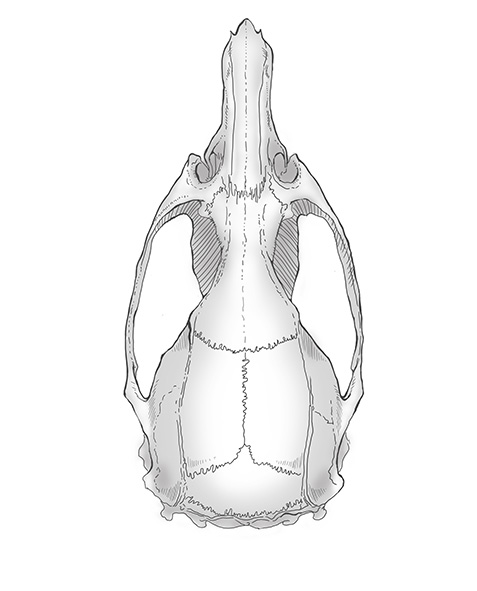

Supplement: Supplemental Information 2 [file peerj-11-16555-s002.zip › figure 1/B.jpg]

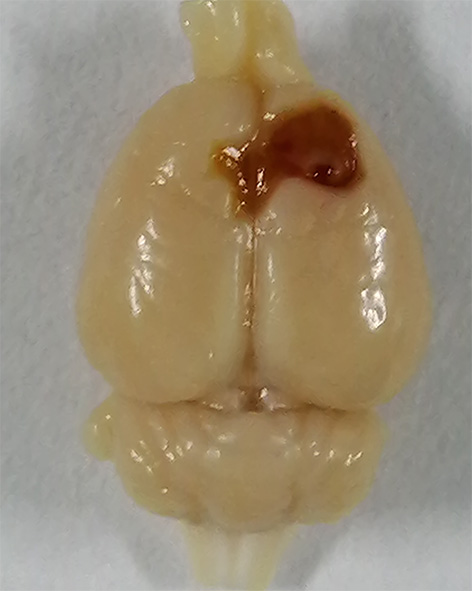

Supplement: Supplemental Information 2 [file peerj-11-16555-s002.zip › figure 1/C.jpg]

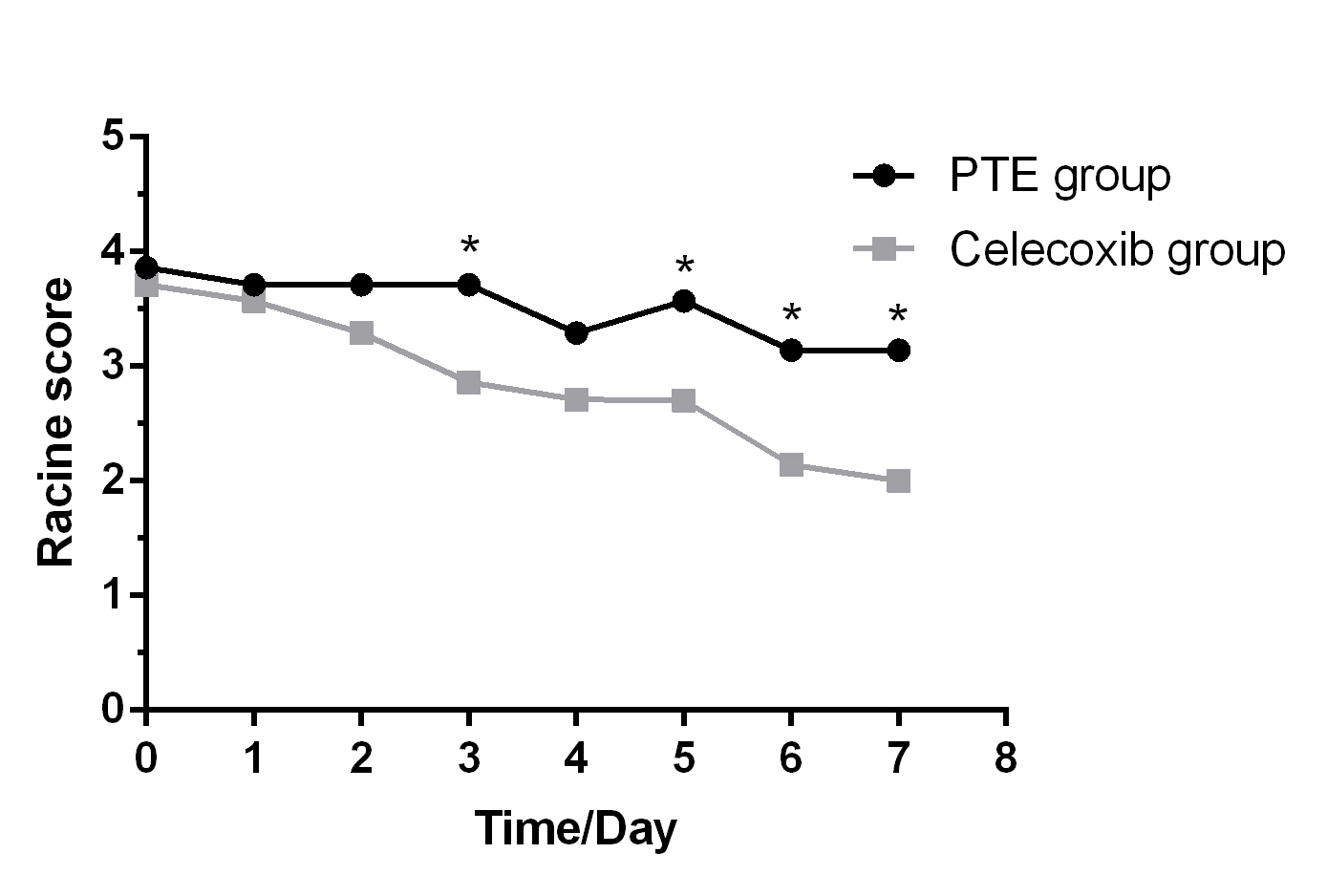

Supplement: Supplemental Information 2 [file peerj-11-16555-s002.zip › figure 2/Figure 2.jpg]

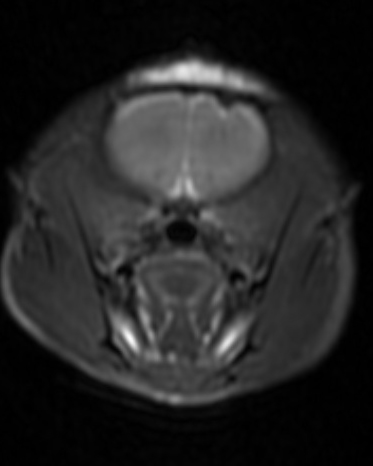

Supplement: Supplemental Information 2 [file peerj-11-16555-s002.zip › figure 3/MRI image on 7th day/Celecoxib/IMG-0004-00001.jpg]

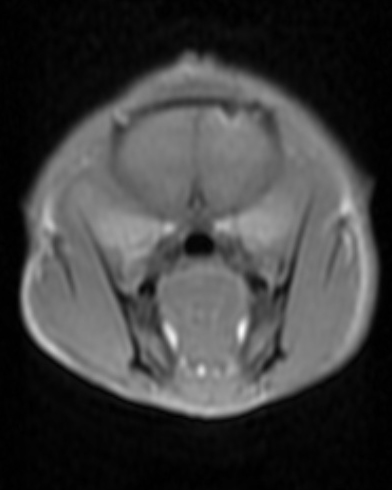

Supplement: Supplemental Information 2 [file peerj-11-16555-s002.zip › figure 3/MRI image on 7th day/Celecoxib/IMG-0005-00001.jpg]

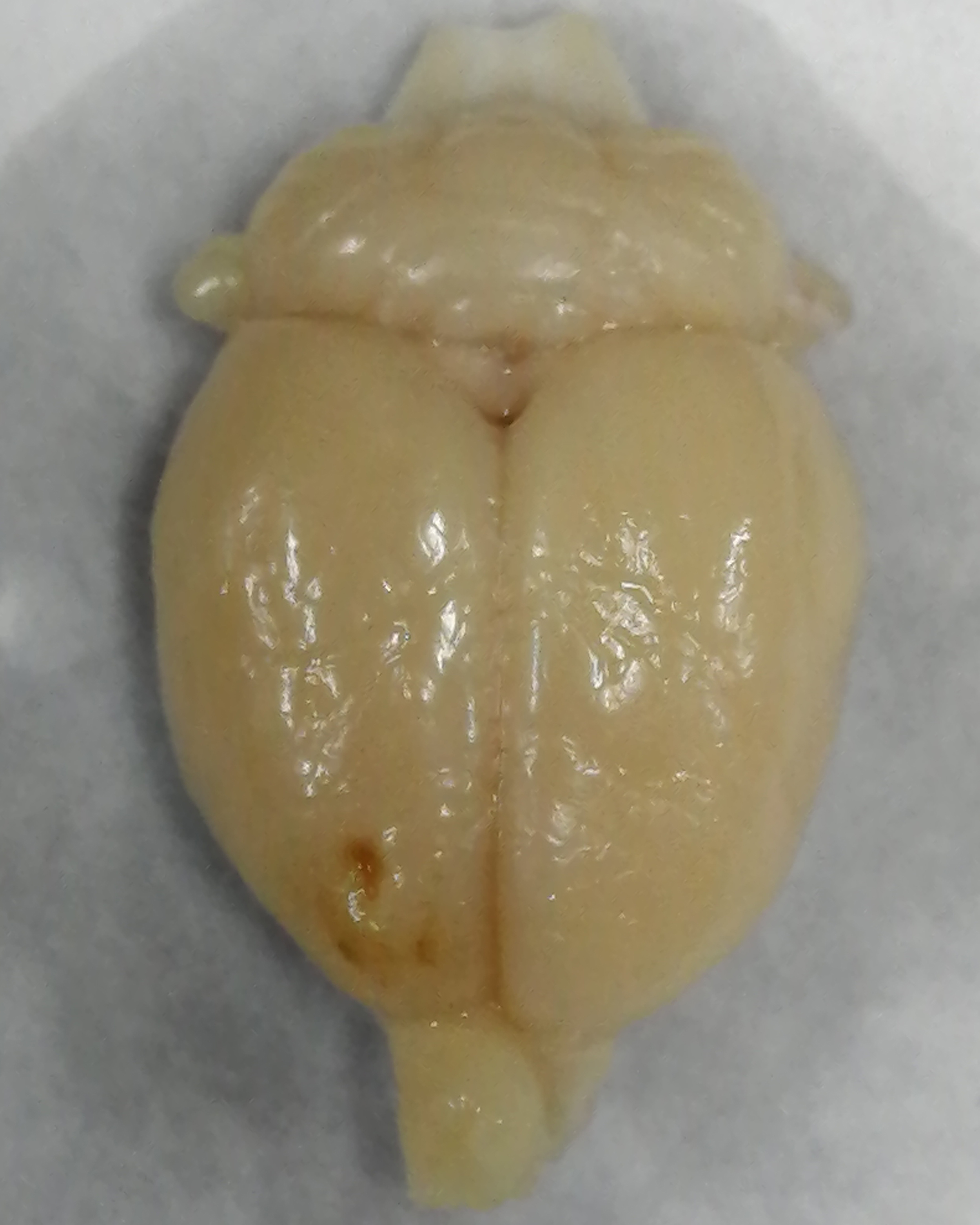

Supplement: Supplemental Information 2 [file peerj-11-16555-s002.zip › figure 3/MRI image on 7th day/Celecoxib/╬ó╨┼═╝╞1⁄4_20210103221806.jpg]

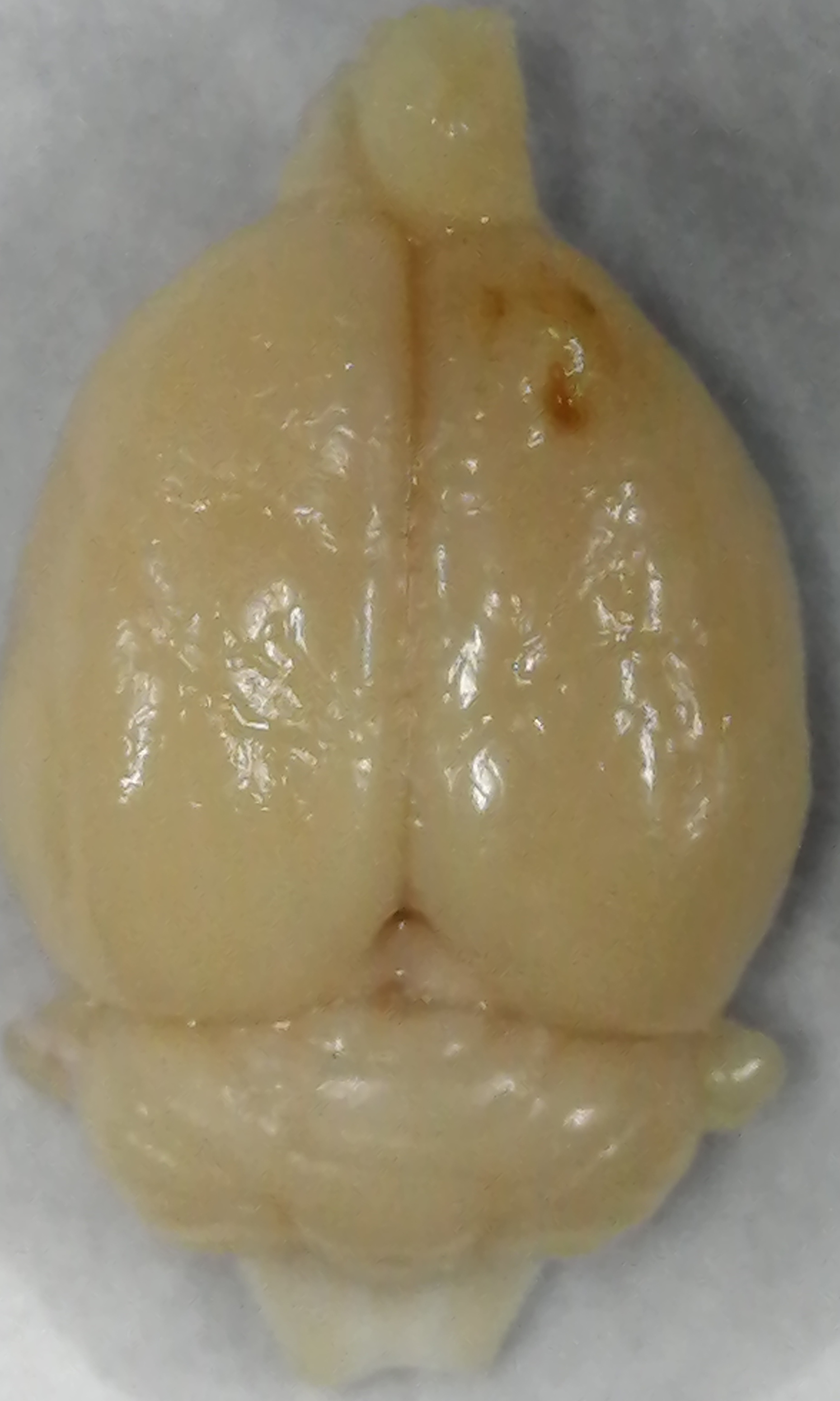

Supplement: Supplemental Information 2 [file peerj-11-16555-s002.zip › figure 3/MRI image on 7th day/Celecoxib/╬ó╨┼═╝╞1⁄4_20210103221806╙├.jpg]

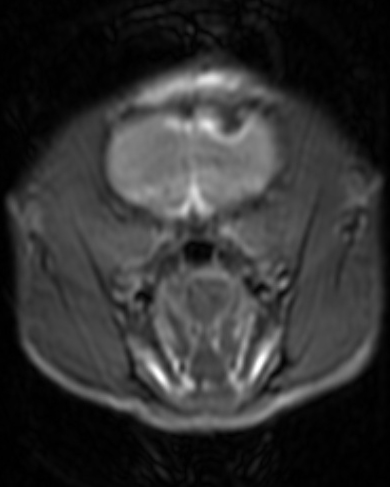

Supplement: Supplemental Information 2 [file peerj-11-16555-s002.zip › figure 3/MRI image on 7th day/PTE/IMG-0006-00001.jpg]

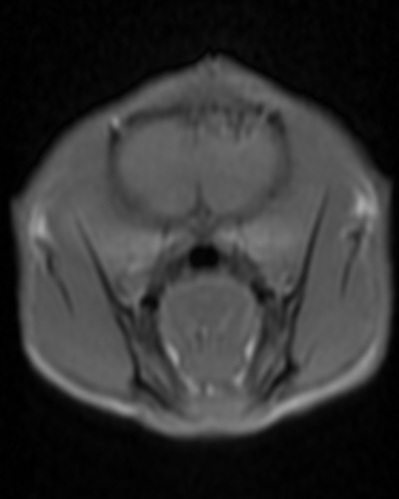

Supplement: Supplemental Information 2 [file peerj-11-16555-s002.zip › figure 3/MRI image on 7th day/PTE/IMG-0007-00002.jpg]

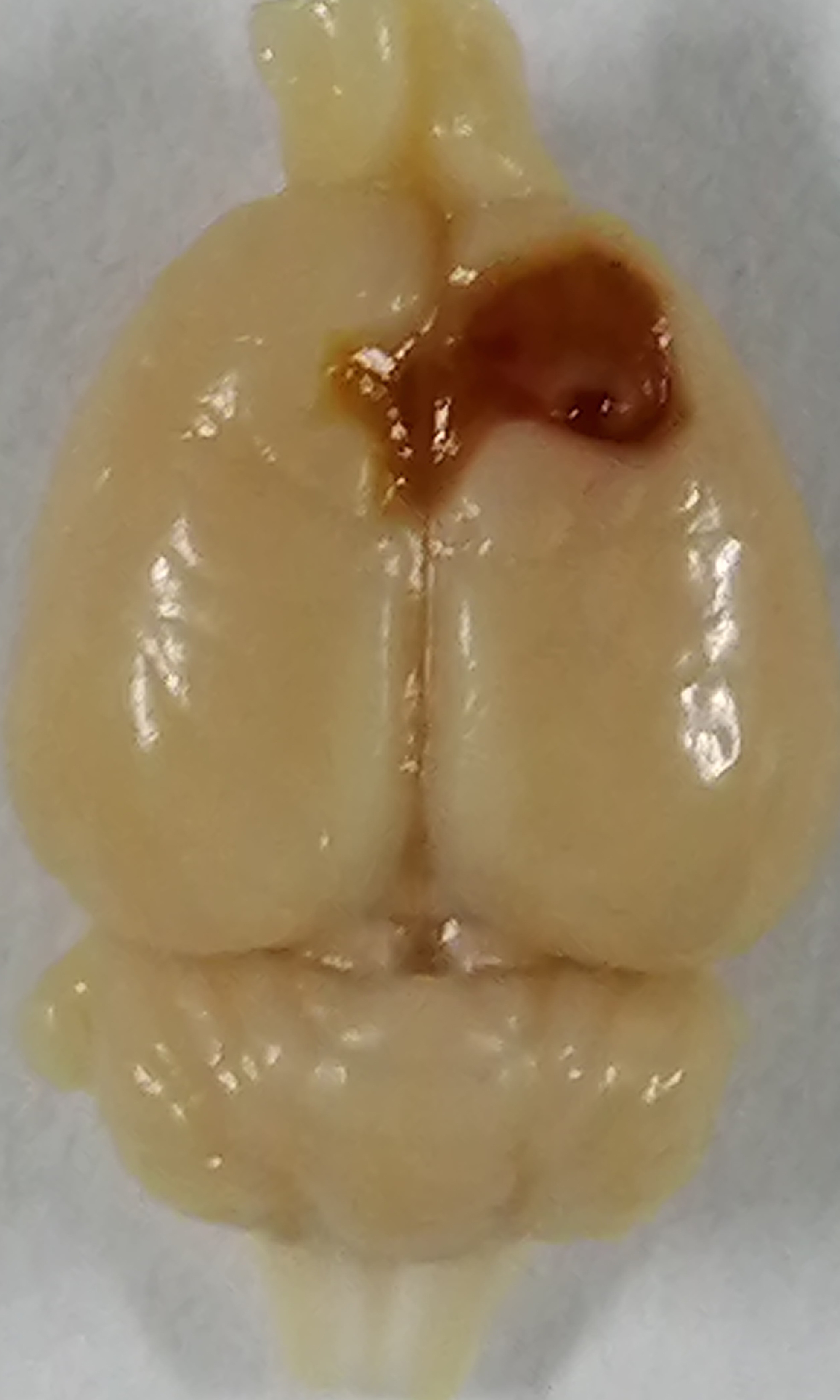

Supplement: Supplemental Information 2 [file peerj-11-16555-s002.zip › figure 3/MRI image on 7th day/PTE/╬ó╨┼═╝╞1⁄4_20210103222533╙├.jpg]

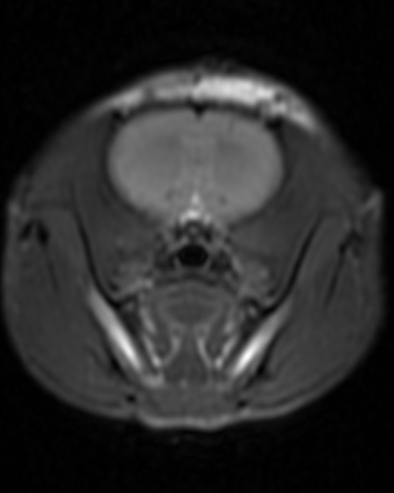

Supplement: Supplemental Information 2 [file peerj-11-16555-s002.zip › figure 3/MRI image on 7th day/Sham/IMG-0008-00001.jpg]

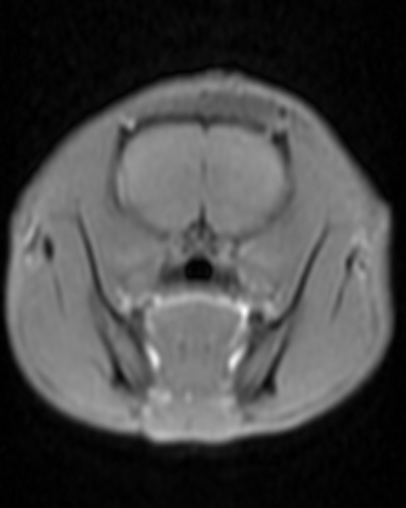

Supplement: Supplemental Information 2 [file peerj-11-16555-s002.zip › figure 3/MRI image on 7th day/Sham/IMG-0009-00001.jpg]

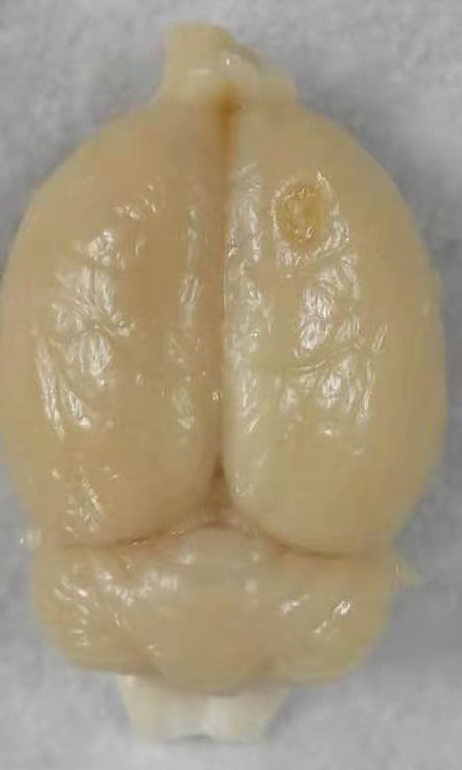

Supplement: Supplemental Information 2 [file peerj-11-16555-s002.zip › figure 3/MRI image on 7th day/Sham/╬ó╨┼═╝╞1⁄4_20210308213448╙├.jpg]

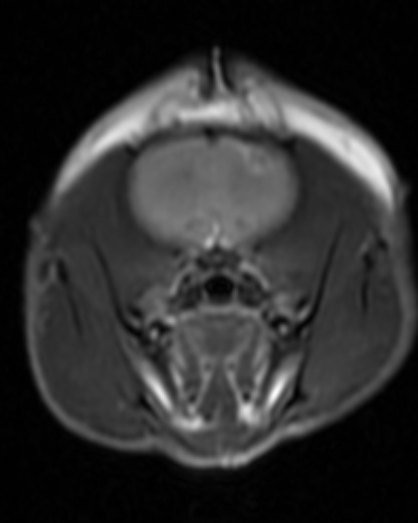

Supplement: Supplemental Information 2 [file peerj-11-16555-s002.zip › figure 3/MRI image on the 3rd day/Celecoxib/IMG-0007-00001.jpg]

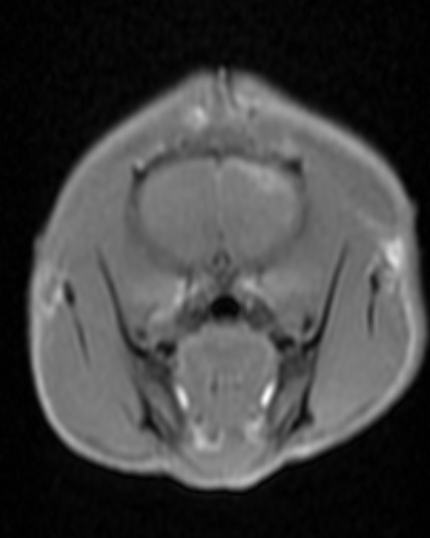

Supplement: Supplemental Information 2 [file peerj-11-16555-s002.zip › figure 3/MRI image on the 3rd day/Celecoxib/IMG-0008-00001.jpg]

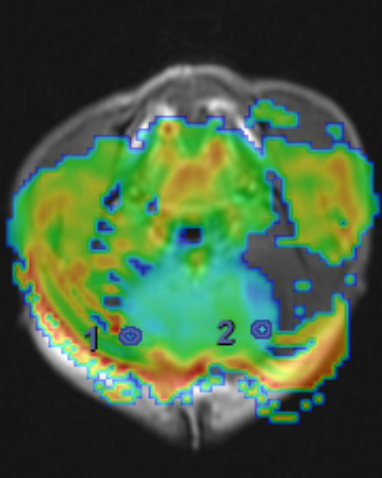

Supplement: Supplemental Information 2 [file peerj-11-16555-s002.zip › figure 3/MRI image on the 3rd day/Celecoxib/IMG-0009-00001.jpg]

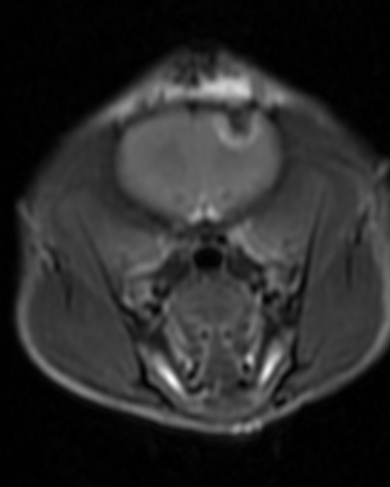

Supplement: Supplemental Information 2 [file peerj-11-16555-s002.zip › figure 3/MRI image on the 3rd day/PTE/IMG-0001-00001.jpg]

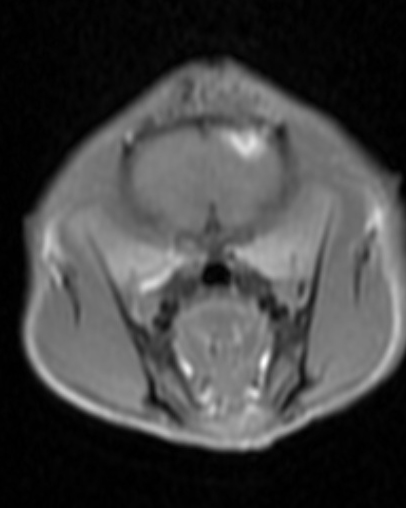

Supplement: Supplemental Information 2 [file peerj-11-16555-s002.zip › figure 3/MRI image on the 3rd day/PTE/IMG-0002-00002.jpg]

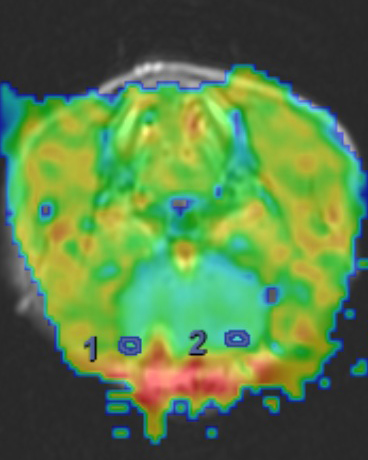

Supplement: Supplemental Information 2 [file peerj-11-16555-s002.zip › figure 3/MRI image on the 3rd day/PTE/IMG-0005-00001.jpg]

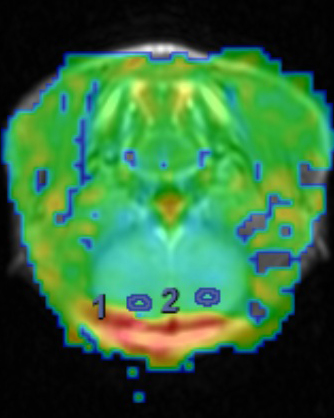

Supplement: Supplemental Information 2 [file peerj-11-16555-s002.zip › figure 3/MRI image on the 3rd day/Sham/IMG-0010-00001.jpg]

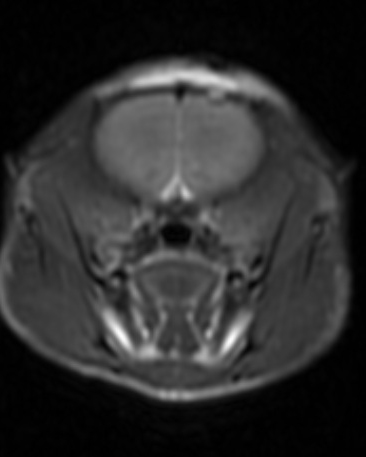

Supplement: Supplemental Information 2 [file peerj-11-16555-s002.zip › figure 3/MRI image on the 3rd day/Sham/IMG-0011-00001.jpg]

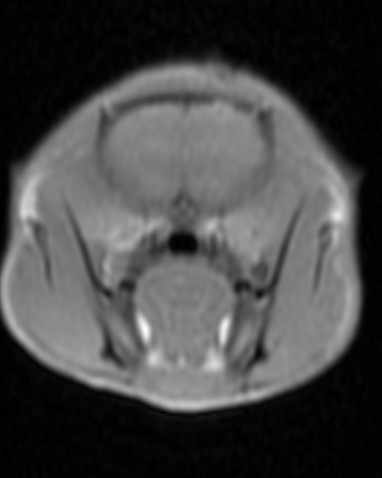

Supplement: Supplemental Information 2 [file peerj-11-16555-s002.zip › figure 3/MRI image on the 3rd day/Sham/IMG-0012-00001.jpg]

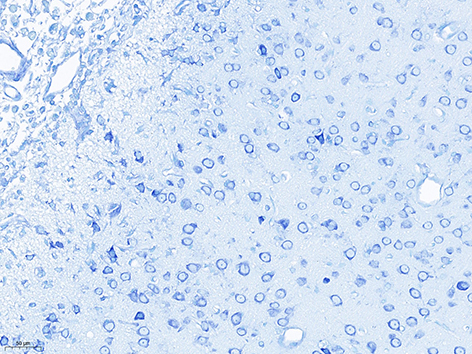

Supplement: Supplemental Information 3 [file peerj-11-16555-s003.zip › figure 4/Celecoxib .jpg]

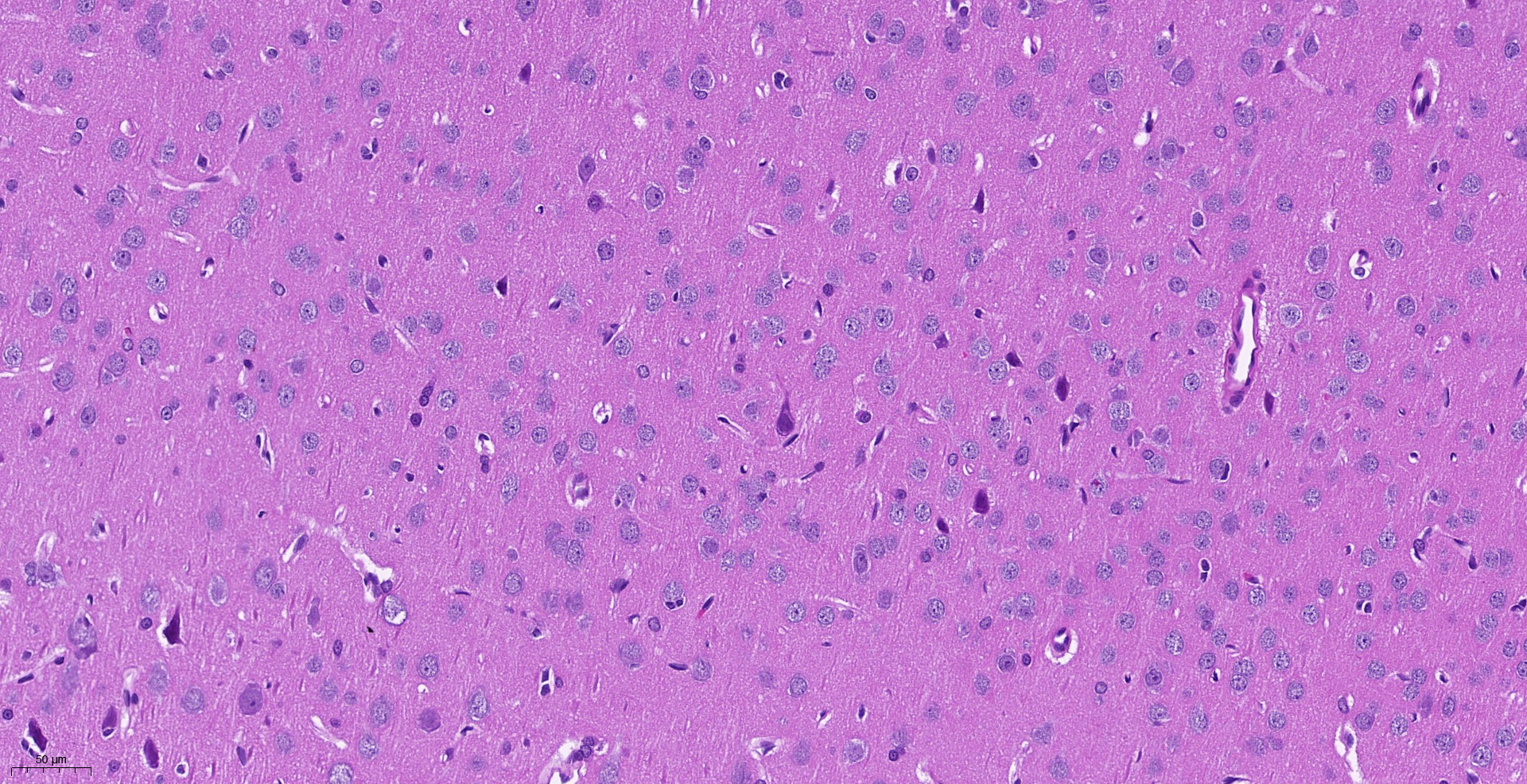

Supplement: Supplemental Information 3 [file peerj-11-16555-s003.zip › figure 4/Celecoxib HE.jpg]

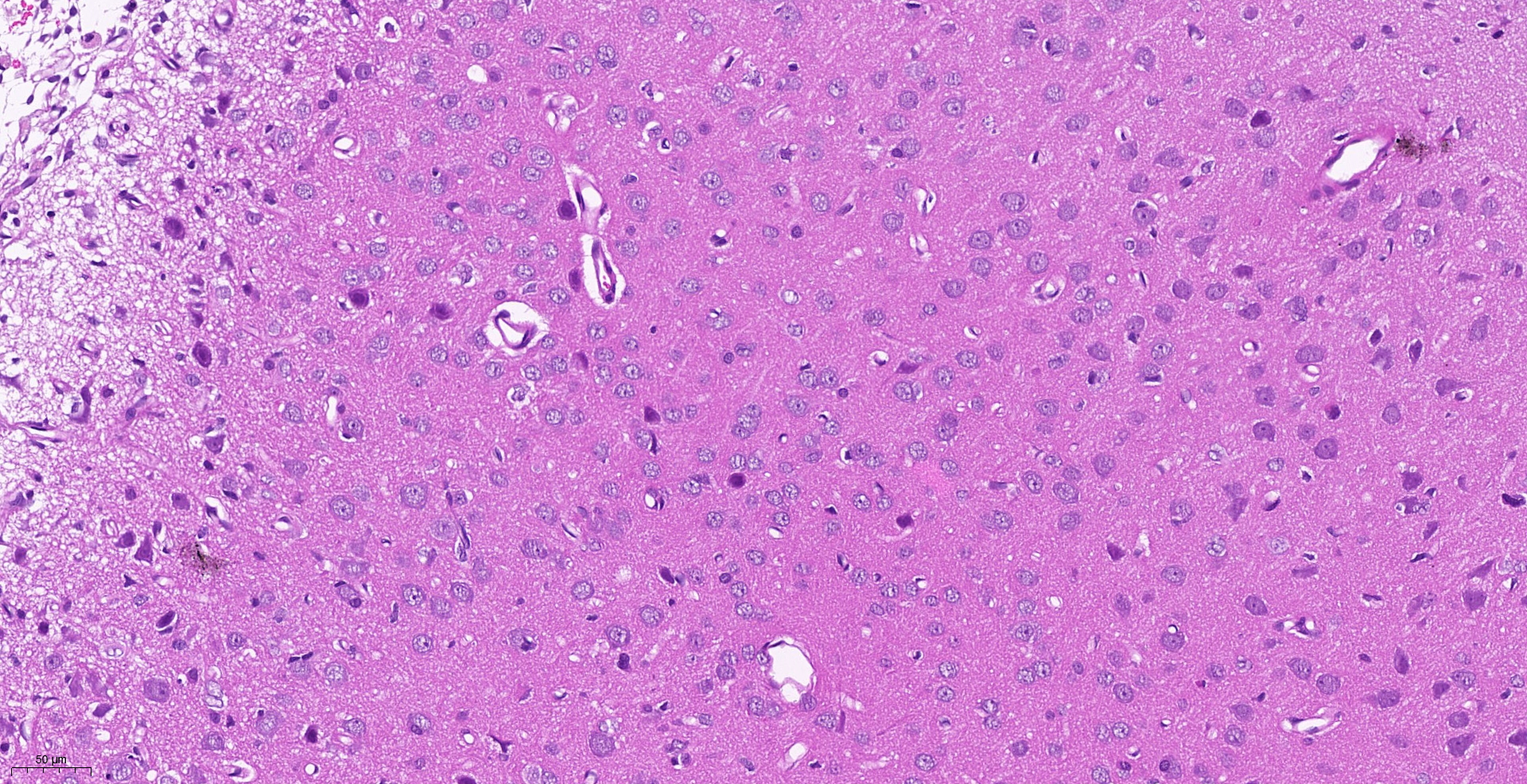

Supplement: Supplemental Information 3 [file peerj-11-16555-s003.zip › figure 4/PTE HE.jpg]

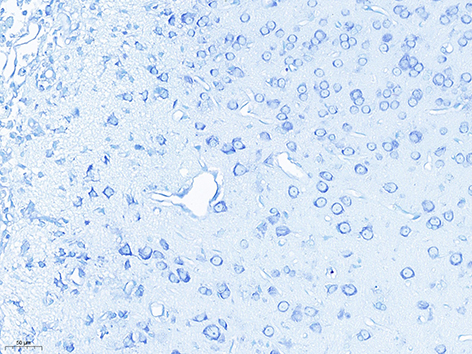

Supplement: Supplemental Information 3 [file peerj-11-16555-s003.zip › figure 4/PTE.jpg]

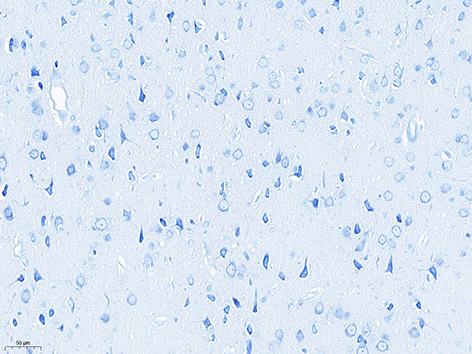

Supplement: Supplemental Information 3 [file peerj-11-16555-s003.zip › figure 4/Sham.jpg]

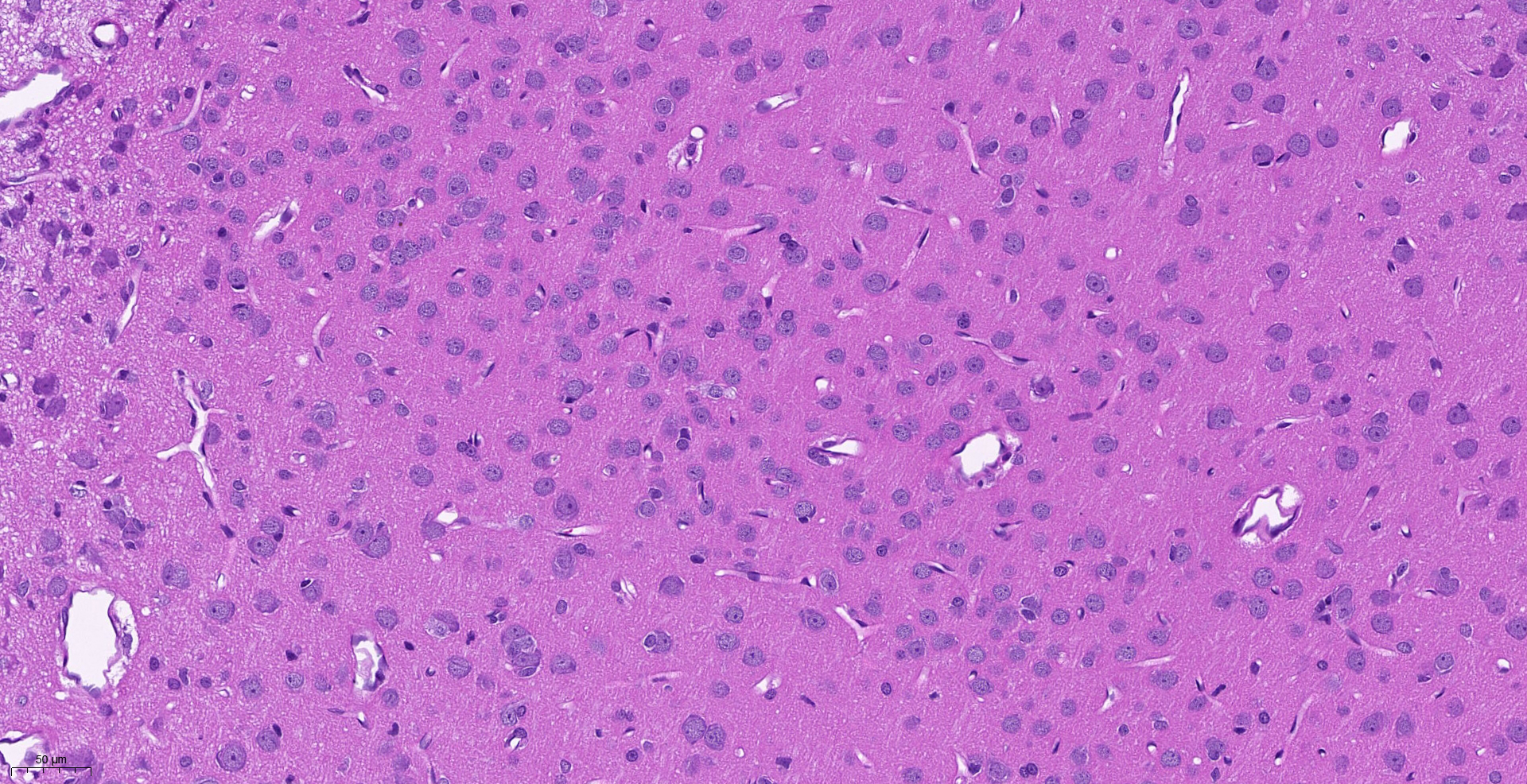

Supplement: Supplemental Information 3 [file peerj-11-16555-s003.zip › figure 4/Sham-HE.jpg]

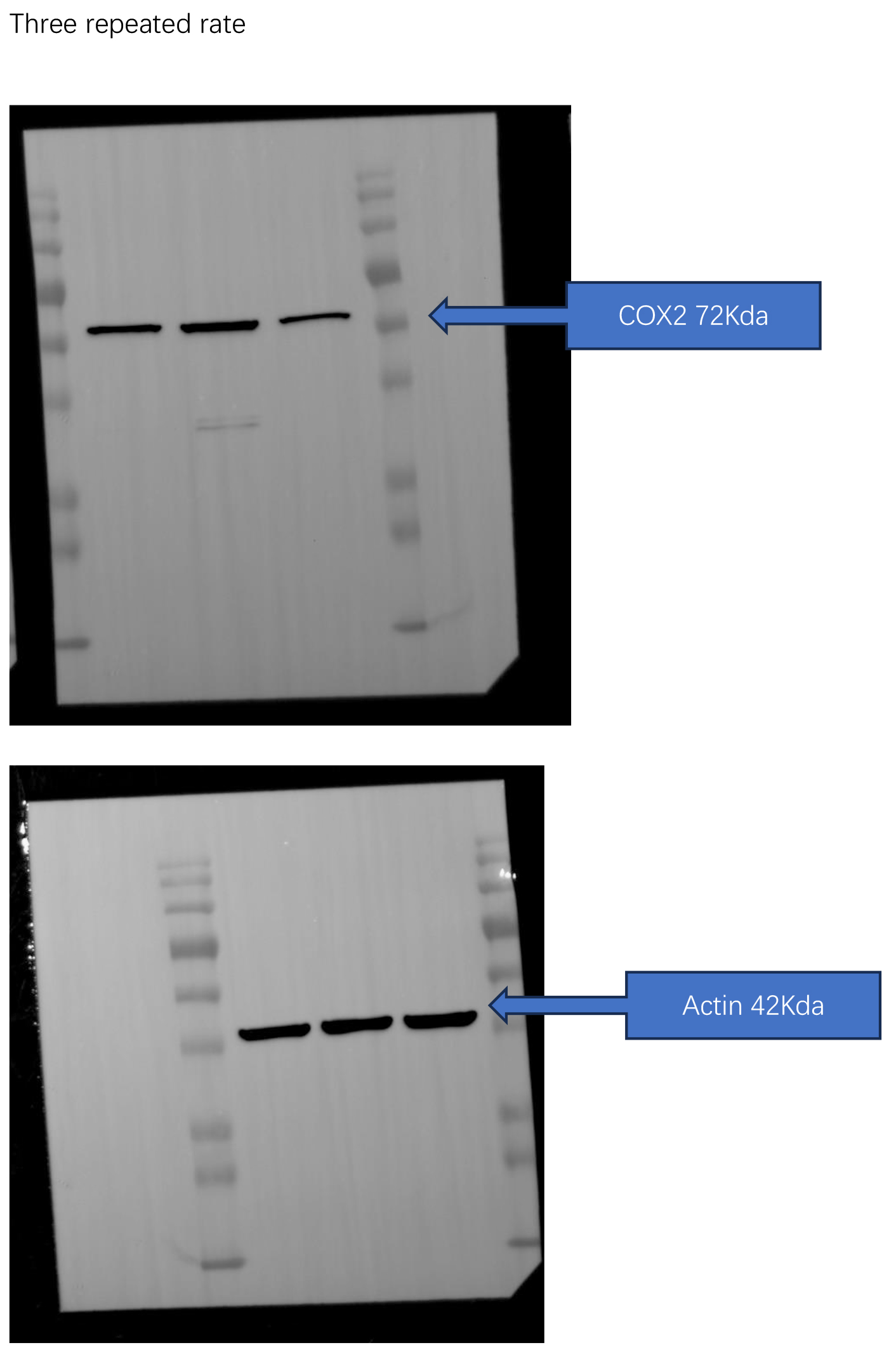

Supplement: Supplemental Information 3 [file peerj-11-16555-s003.zip › figure 5/original date-1.jpg]

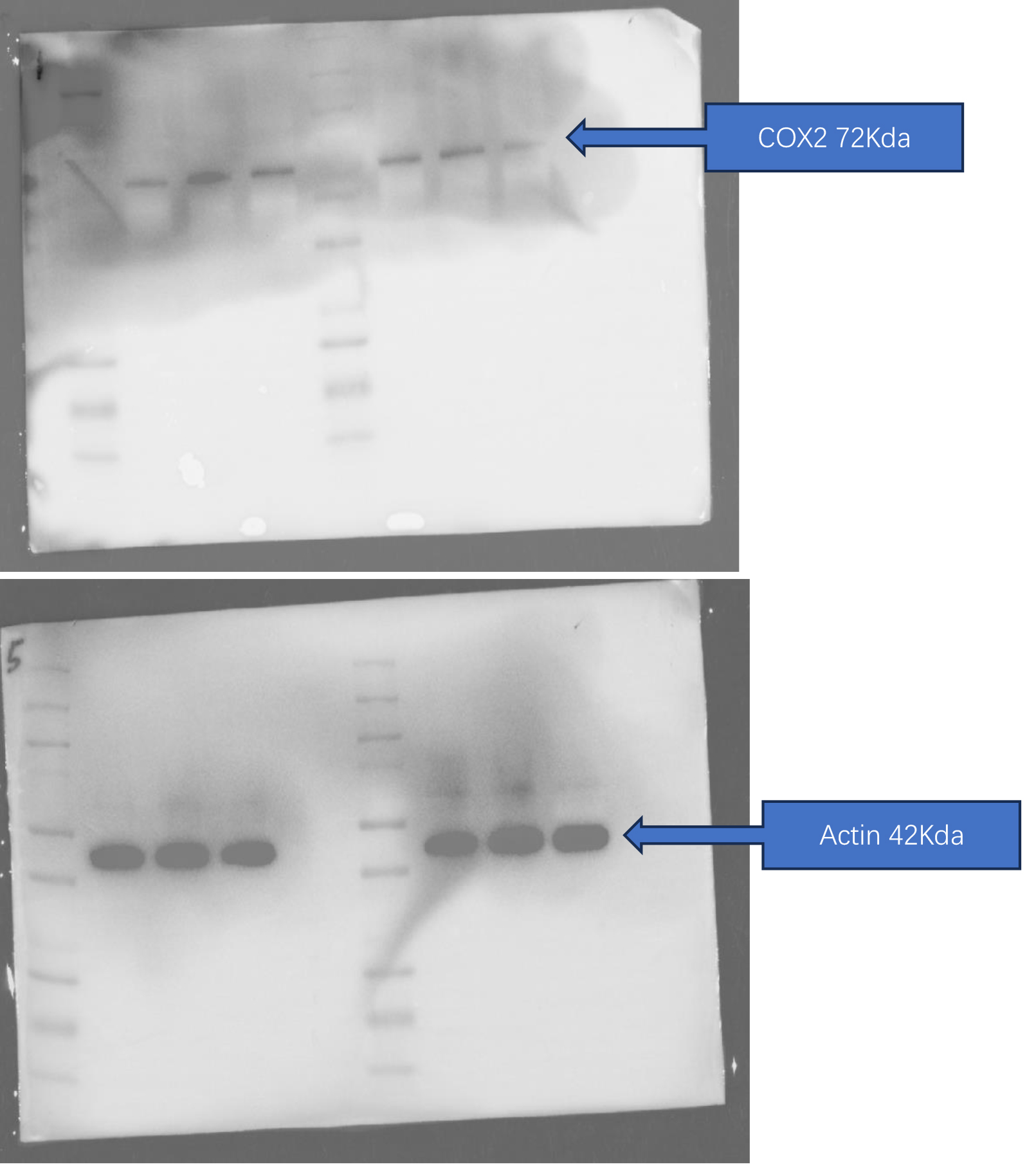

Supplement: Supplemental Information 3 [file peerj-11-16555-s003.zip › figure 5/original date-2.jpg]
